# Platelet function and filamin A expression in two families with novel *FLNA* gene mutations associated with periventricular nodular heterotopia and panlobular emphysema

**DOI:** 10.1002/ajmg.a.62690

**Published:** 2022-02-14

**Authors:** Laura M. Tanner, Shinji Kunishima, Elina Lehtinen, Tuukka Helin, Kirsi Volmonen, Riitta Lassila, Minna Pöyhönen

**Affiliations:** ^1^ HUSLAB Department of Clinical Genetics Helsinki University Hospital Helsinki Finland; ^2^ Department of Medical and Clinical Genetics University of Helsinki Helsinki Finland; ^3^ Department of Medical Technology Gifu University of Medical Science Gifu Japan; ^4^ Coagulation Disorders Unit Helsinki University Hospital, Research Program Unit in Systems Oncology, University of Helsinki Helsinki Finland; ^5^ HUSLAB Department of Chemistry and Microbiology Helsinki University Hospital Helsinki Finland; ^6^ HUS Medical Imaging Center Helsinki University Hospital Helsinki Finland

**Keywords:** filamin A, panlobular emphysema, periventricular nodular heterotopia, platelet function test, thrombocytopenia

## Abstract

Pathogenic variants of the X‐linked FLNA gene encoding filamin A protein have been associated with a wide spectrum of symptoms, including the recently described pulmonary phenotype with childhood‐onset panlobular emphysema. We describe three female patients from two families with novel heterozygous FLNA variants c.5837_2del and c.508C > T. Analysis of immunofluorescence of peripheral blood smears and platelet function was performed for all patients. FLNA‐negative platelets were observed, suggesting that these variants result in the loss of a functional protein product. All three patients also had periventricular nodular heterotopia and panlobular emphysema. However, they had considerably milder symptoms and later age of onset than in the previously reported cases. Therefore, patients with pathogenic FLNA variants should be studied actively for lung involvement even in the absence of pronounced respiratory symptoms. Conversely, any patient with unexplained panlobular emphysema should be analyzed for pathogenic FLNA variants. We also suggest that immunofluorescence analysis is a useful tool for investigating the pathogenicity of novel FLNA variants.

## 1 INTRODUCTION

The X‐linked *FLNA* gene (OMIM 300017) encodes filamin A, a F‐actin‐binding cytoplasmic phosphoprotein involved in cytoskeletal remodeling of cell morphology and migration. Pathogenic variants of the *FLNA* gene cause a wide spectrum of rare diseases called filaminopathies A. Heterozygous truncating variants primarily result in periventricular nodular heterotopia (PVNH), a brain disorder in which neuronal migration to the cortex is disrupted during early fetal development (OMIM #300049). Additional characteristics of *FLNA*‐related PVNH include megacisterna magna, cardiovascular malformations, epilepsy and Ehlers–Danlos‐like collagen defects. It is mostly encountered in heterozygous females, as truncating variants are usually lethal in males (Parrini et al., 2006; Sheen et al., 2005). Gain‐of‐function variants, on the other hand, cause a variety of congenital malformation syndromes including otopalatodigital spectrum disorders (OMIM #304120, OMIM #311300), frontometaphyseal dysplasia (OMIM #305620), and Melnick–Needles syndrome (OMIM #309350) (Robertson et al., 2003; Wade et al., 2020). Surviving males with hemizygous pathogenic *FLNA* variants generally present with severe phenotypes (Cannaerts et al., 2018; Ritelli et al., 2017; Spencer et al., 2018). However, several male patients have been reported to survive even to adulthood by Iqbal et al; these patients had a distinct phenotype with prune belly syndrome with or without additional findings associated with otopalatodigital spectrum disorder (Iqbal et al., 2020). The link between pathogenic FLNA variants and congenital lung disease was first suggested by Masurel‐Paulet et al., 2011. Recently, several cases with a childhood‐onset respiratory phenotype characterized by pulmonary emphysema, atelectasis, and pulmonary vascular attenuation/malformation have been described (Eltahir et al., 2016; Pelizzo et al., 2019; Sasaki et al., 2019; Shelmerdine et al., 2017).

Abnormal platelet morphology and function have been described in several patients with FLNA‐associated disease and Nurden et al. have suggested that macrothrombocytopenia may even present as the primary phenotype (Nurden et al., 2011). As blood platelets are easily available for isolation, their structural and functional analysis may provide useful data on the pathogenicity of novel *FLNA* variants. Ieda et al. have suggested immunofluorescence staining of peripheral blood smears focusing on platelets as a convenient method for identifying patients with FLNA variants (Ieda et al., 2018). *FLNA* is expressed in platelets and its pathogenic variants alter platelet production from the bone marrow megakaryocytes, leading to reduced number and abnormal size of platelets (Rosa et al., 2019). Lopez et al. have also suggested that deficient expression or function of filamin A might lead to altered Ca^2+^ homeostasis in platelets and, subsequently, functional abnormalities (Lopez et al., 2018).

We describe here two novel *FLNA* variants associated with periventricular heterotopia and pulmonary emphysema, with respective alterations observed in platelet function and *FLNA* expression.

## 2 PATIENTS AND METHODS

### 2.1 Patients

#### 2.1.1 Family 1

**FIGURE 1:**
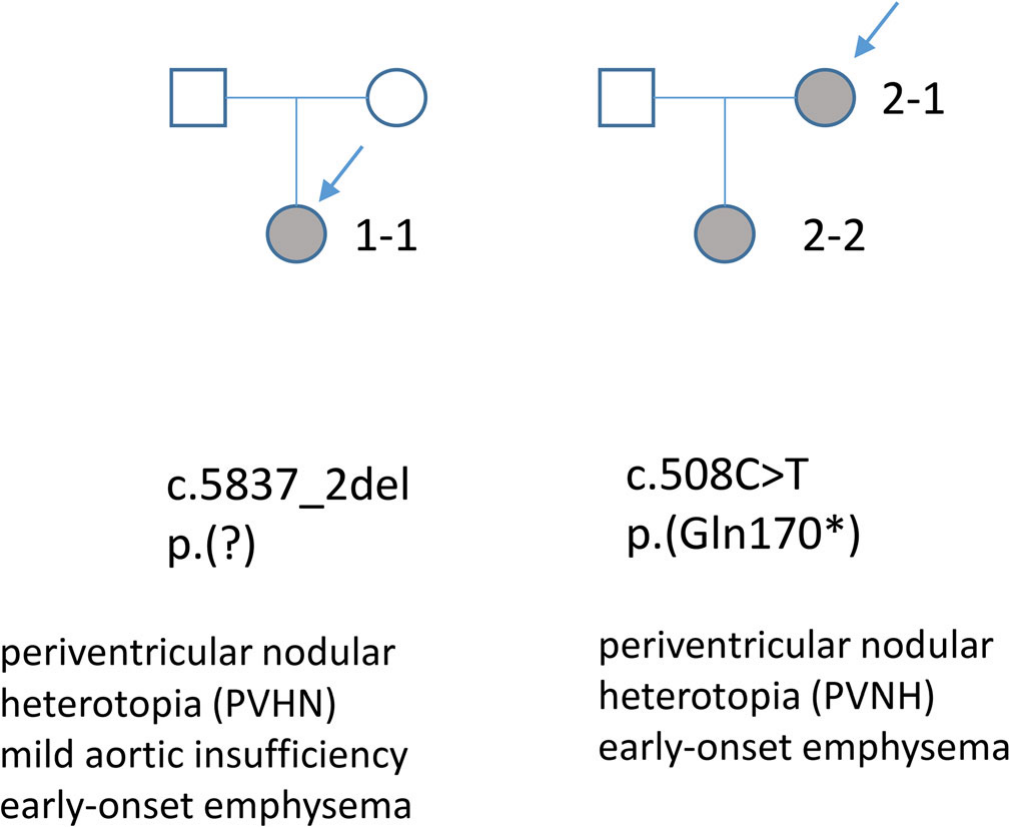
Pedigrees of the two families with novel FLNA variants

**FIGURE 2:**
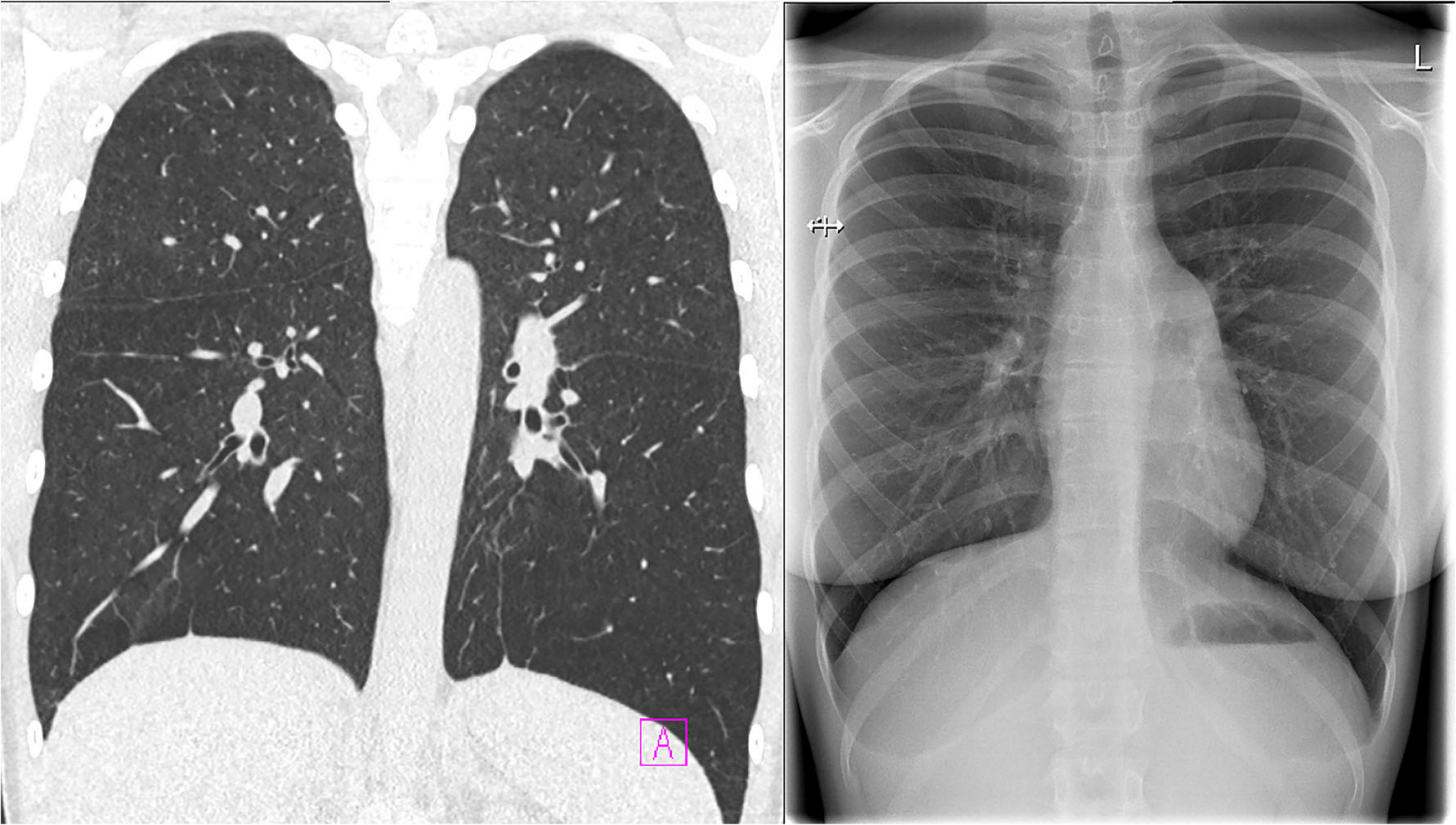
Contrast enhanced coronal chest CT (left) and X‐ray (right) images with panlobular emphysema in patient 1–1

The patient (1–1) (Figure 1) is a young woman who had her first epileptic seizure at 14 years of age. Subsequent brain MRI revealed periventricular nodular heterotopia and megacisterna magna, typical findings of *FLNA*‐related PVNH. She has otherwise been well, with normal growth pattern and cognitive development. However, she does have mild joint laxity, bilateral pes planus, mild aortic valve regurgitation, and persisting mild thrombocytopenia without bleeding symptoms (132–158 E9/L, reference range 200–350 E9/L). At 15 years of age, her aortic diameter was found to be slightly increased but within the normal limits (largest diameter 28 mm). A heterozygous de novo *FLNA* variant c.5837‐2del p.(?) was detected with a targeted next generation sequencing (NGS) gene panel (reference sequence NM_001456.3, genomic description NC_000023.11:g.154353459del). This variant results in a bioinformatically predicted loss of a splice acceptor site. To our knowledge, this variant has not been previously reported in the literature nor listed in gnomAD, ClinVar, or HGMD databases. After the diagnosis, thorough functional and imaging studies were performed to rule out pulmonary manifestations, although the patient did not have any history of dyspnea or asthma. Surprisingly, she was found to have markedly decreased total lung diffusion capacity (65%) and lung CT revealed panlobular emphysema (Figure 2).

#### 2.1.2 Family 2

**FIGURE 3:**
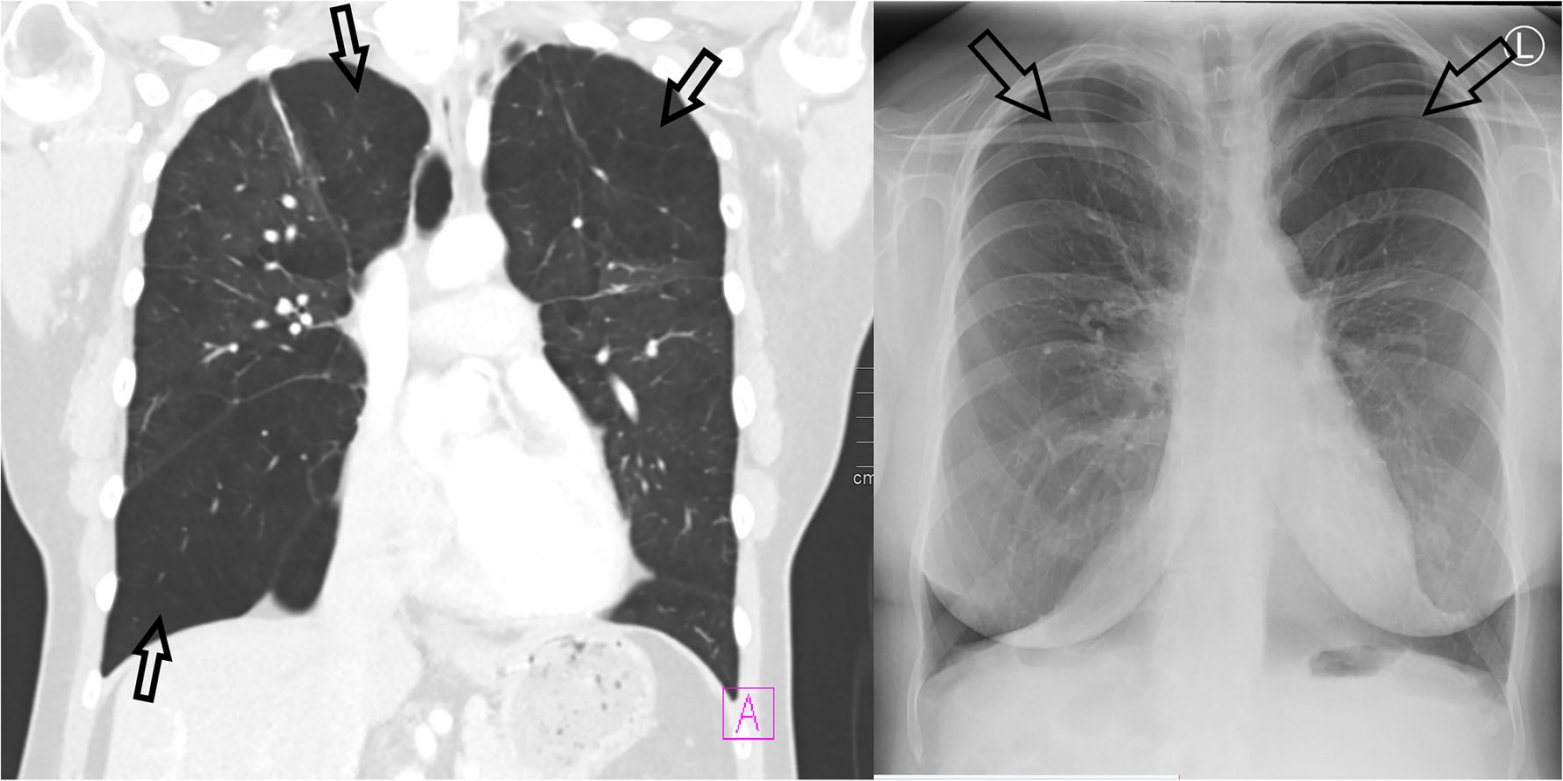
Contrast enhanced coronal chest CT (left) and X‐ray (right) images with marked panlobular emphysema in patient 2–1. There is abnormal hyperlucency of both lungs with paucity of the peripheral lung vessels (arrows)

The index patient (2–1) is a 40‐year‐old woman with diagnoses of asthma and recurrent pulmonary infections since childhood. At the age of 37 years she was diagnosed with panlobular emphysema without history of cigarette smoking or alpha‐1‐antitrypsin deficiency (Figure 3). She also had mild joint hypermobility, PVNH, and a history of recurrent miscarriages. Targeted NGS gene panel revealed a novel likely pathogenic *FLNA* variant c.508C > T p.(Gln170*), and also another variant of unknown significance c.1933G > A p.(Val645Ile) (reference sequence NM_001110556.1). These two variants were found *in cis*, and the latter variant is located downstream from the premature stop codon created by the c.508C > T variant. It is therefore unlikely that this variant would have any clinical significance. The variant c.508C > T has not been previously reported in the literature or gnomAD, ClinVar, or HGMD databases.

The index patient has one 15‐year‐old daughter (2–2), who carries both of the *FLNA* variants detected in her mother. She was diagnosed with asthma in early childhood and has been suffering from exercise‐induced dyspnea responding poorly to medication but has otherwise been doing well. Diagnostic work‐up again revealed panlobular emphysema, mild joint hypermobility, and PVNH.

### 2.2 Methods

Written informed consent was obtained from all patients or their guardians for the platelet immunofluorescence and functional analysis. Other analyses including genetic testing and radiological imaging were performed as parts of our routine diagnostic protocol. Novel gene variants have been submitted to the Leiden Open Variation Database https://databases.lovd.nl/shared/genes/FLNA.

Peripheral blood smears were analyzed by immunofluorescence staining as described by Oda et al., 2016. Samples from family members not carrying the detected *FLNA* variant were used as controls. For platelet function analyses, we performed the Innovance PFA‐200® (platelet function analyzer, PFA) assay (Siemens Healthcare). 3.2% Na‐citrate‐anticoagulated whole blood samples were subjected to agonists of epinephrine and ADP in interaction with collagen under flow conditions. The PFA is a platelet function screening test with a high sensitivity (Koscielny et al., 2004). Platelet impedance aggregometry in whole blood by using Multiplate® analyzer (Roche) was also performed (Moenen et al., 2019). In the Multiplate® assay, platelet function can be further characterized using different agonists. Hirudin‐anticoagulated blood (25 μg/ml) was run with the following agonists and concentrations: ristocetin 1.2 mg/ml, 0.6 mg/ml, and 0.3 mg/ml, ADP 6.5 μM, ADP 2.0 μM arachidonic acid (ASPI) 0.5 mM, thrombin receptor activating peptide (TRAP) 32 μM and collagen 3.2 μg/ml. In addition, ristocetin at concentrations 1.2, 0.6 and 0.3 mg/ml were added to 3.2% citrated blood as well, per our routine protocols. Only the results of ristocetin 1.2 mg/ml (RISTOH), ADP 6.5 mM, ASPI 0.5 mM, TRAP 32 μM in Hirudin‐anticoagulated blood are routinely reported, the other abovementioned assay results are reported only if abnormal. We chose these assays for platelet function analyses since they are performed routinely in our laboratory. They are analyzed in whole blood, facilitating sample preparation and improving workflow.

In order to screen for coagulation defects as well, a panel from 3.2% Na‐citrated plasma was performed, including prothrombin time (PT), activated partial thromboplastin time (APTT), thrombin time (TT), von Willebrand factor (VWF) activity using platelet glycoprotein (GP)Ib binding (VWF:GPIbM), VWF antigen (VWF:Ag), fibrinogen, coagulation factor (F)V, FVIII, FIX, and FXIII. In addition, FII and FX were analyzed if PT was abnormal (patient 1–1). The assays were performed by using Instrumentation Laboratory ACT TOP 500® and Siemens Healthcare BCS XP® analyzers and the corresponding reagents.

Bleeding symptoms were assessed with ISTH/SSC bleeding assessment tool (bleedingscore.certe.nl).

## 3 RESULTS

Results are shown in Table 1

**TABLE 1:** Laboratory results of the three patients with novel heterozygous FLNA mutations

Patient	1–1	2–1	2–2
Variant	c.5837_2del heteroz^a^	c.508C > T heteroz^b^	c.508C > T heteroz^b^
Age	16	40	15
IF FLNA‐	98/222	5/152	18/153
IF FLNA‐%	44.1	3.30	11.8
BSCORE	0	8	3
B‐PLT	**145**	**157**	**194**
P‐PT	**67**	124	84
B‐RISTOH	76	**68**	95
B‐RISTO 0.6	**37**	61	84
B‐ADP	73	68	77
B‐ASPI	101	85	105
B‐TRAP	129	122	119
B‐CO/EPI1	**>300**	94	112
B‐CO/EPI2	**>300**	101	129
B‐CO/EPI3	ND	ND	ND
B‐CO/ADP1	**199**	94	**108**
B‐CO/ADP2	**199**	87	**112**
B‐CO/ADP3	ND	ND	ND
P‐APTT	33	29	30
P‐TT	23	20	22
P‐FVIII	70	173	141
vWF:GPibM	57	127	111
P‐vWF‐Ag	61	142	117
P‐FIBR	**1.9**	3.6	2
P‐FII	105	ND	ND
P‐FV	84	106	87
P‐FVII	61	114	82
P‐FIX	65	125	95
P‐FX	96	ND	ND
P‐FXI	ND	ND	ND
P‐FXII	ND	ND	ND
P‐FXIII	107	105	82
SMEAR	SV	MM	SV

*Note*: Values outside the age‐ and gender‐dependent reference range have been bolded.

Abbreviations: B‐ADP, response to adenosine diphosphate 6.5 μM; B‐ASPI, response to arachidonic acid 0,5 mM; B‐CO/ADP, response to collagen/ADP; B‐CO/EPI, response to collagen/epinephrine; B‐PLT, blood platelet count (E9/L, reference range 150–360 for adults, 200–450 for children); B‐RISTOH 0.6, response to ristocetin 0.6 mg/ml; B‐RISTOH, response to ristocetin 1.2 mg/ml; SCORE, ISTH‐SSC bleeding score; B‐TRAP, response to thrombin receptor activating peptide 32 μM; IF FLNA‐%, percentage of FLNA‐negative platelets detected with immunofluorescence staining; IF FLNA, number of FLNA‐negative platelets detected with immunofluorescence staining; MM, moderate macrothrombocytopenia; P‐APTT, plasma activated partial thromboplastin time (seconds, reference range 28–37); P‐FIBR, plasma fibrinogen (g/L, reference range 2–4); P‐FII‐XIII, plasma coagulation factors II‐XIII (%); P‐PT, prothrombin time (%, reference range 70–130); P‐TT, plasma thrombin time (seconds, reference range 17–25); SMEAR, peripheral blood smear; SV, increased platelet size variation with occasional macrothrombocytes; vWF:GPibM, von Willebrand factor GPib binding (%, reference range 50–190); vWF‐Ag, von Willebrand factor antigen (%, reference range 50–190).

^a^ Reference sequence NM_001456.3.

^b^ Reference sequence NM_001110556.2.

**FIGURE 4:**
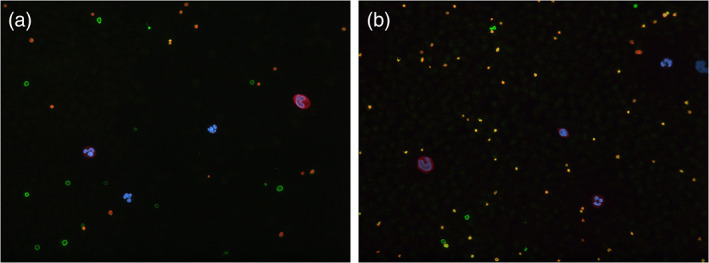
Immunofluorescence staining of peripheral blood of patients 1–1 (a) and 2–2 (b). *FLNA‐*negative platelets only have the beta1‐tubulin positive green control stain whereas platelets with both the beta1‐tubulin positive green control stain and the orange signal positive for filamin A can be seen as yellow

*FLNA‐*negative platelets could be observed in peripheral blood smears of patient 1–1 carrying the FLNA variant c.5837‐2del and patients 2–1 and 2–2 carrying the variant c.508C > T suggesting loss of functional protein. However, the percentage of *FLNA*‐negative platelets was surprisingly low in patients 2–1 and 2–2 (3.3% and 11.8%, respectively). (Figure 4).

Platelet interaction with collagen under blood flow was most sensitive in detecting platelet abnormalities in these patients by providing prolonged closure times under high shear rate flow conditions. Collagen/epinephrine results were grossly abnormal in patient 1–1. Collagen/ADP assessment was abnormal in patient 1–1 and borderline abnormal in patient 2–2. The mild thrombocytopenia is insufficient to explain the PFA findings, but instead a platelet adhesion and aggregation defect on collagen is likely, even in the presence of co‐activation with epinephrine or ADP and shear forces. In contrast, Multiplate aggregation dissociated with the PFA findings, providing essentially normal results, with tight duplicate values. However, in patient 1–1 ristocetin response was diminished at low concentration but normal at the highest ristocetin concentration, suggesting an impaired interaction with platelet glycoprotein Ibα with von Willebrand factor to trigger the signaling.

Coagulation panel was essentially normal in all, with minor inconsequential findings (prolonged PT with marginally low fibrinogen) in patient 1–1.

## 4 DISCUSSION

In this case series, we were able to prove with immunofluorescence analysis that heterozygous variants c.5837_2del and c.508C > T cause loss of functional filamin A protein in platelets.

Childhood‐onset emphysematous lung disease has only recently been added to the spectrum of FLNA‐associated phenotypes. The clinical phenotype has been variable, but several cases requiring lung transplantation have been reported (Burrage et al., 2017). Pelizzo et al., 2020 also recently reported a case where mesenchymal stromal cell infusions were successfully used as a rescue therapy in a child with life‐threatening respiratory syndrome associated with a pathogenic FLNA variant. FLNA‐associated lung disease has typically been diagnosed in the neonatal period or in early childhood (Sasaki et al., 2019). In our patient cohort, one patient was diagnosed with panlobular emphysema in adulthood and two patients in their teens, further broadening the phenotypic spectrum of pathogenic FLNA variants. Computed tomography was required to obtain the correct diagnosis, and in two cases it was only triggered by the available results of the genetic analysis. Pulmonary emphysema is extremely rare in children and even young adults. The most prevalent forms of pulmonary emphysema are smoking‐related centrilobular and paraseptal emphysema. In contrast, panlobular emphysema is typically associated with alpha‐1‐antitrypsin deficiency (Smith et al., 2014).

Majority of patients with *FLNA*‐associated pulmonary disease have had PVNH and/or aortic dilatation (Burrage et al., 2017; Sasaki et al., 2019). On the other hand, in a cohort of 114 patients with FLNA‐associated PVNH described by Chen et al., 2018 21 patients (18.4%) were diagnosed with thoracic aortic aneurysm. Six of them required surgical repair and two additional patients, who did not fulfill the clinical criteria for surgical correction, nevertheless died of spontaneous aortic rupture. Indeed, the importance of screening must be emphasized for all patients with pathogenic FLNA variants both for aortic dilatation and interstitial pulmonary disease.

In our case series, pathogenic loss‐of‐function *FLNA* variants were associated with abnormal platelet interactions under high shear rate blood flow conditions, compatible with the platelet cytoskeletal defects altering adhesive functions toward standardized collagen. As to the modest bleeding tendency and quantity of *FLNA*‐negative platelets, the severity of the overall clinical phenotype makes skewed X‐inactivation an unlikely explanation. It is likely that *FLNA*‐negative megakaryocytes produce abnormal platelets with shortened lifespan, as suggested by Begonja et al., 2011. Majority of the circulating platelets would therefore have wild‐type DNA.

Truncating *FLNA* variants have been reported by Berrou et al. to cause abnormal platelet responses to collagen impairing aggregation, secretion, thrombus formation and GPVI signaling (Berrou et al., 2013). Yet none of our patients exhibited abnormal response to collagen in the Multiplate assay, all having some expression of *FLNA*. We did not have light transmission aggregometry (LTA) results from these patients; however, recently the Multiplate assay had excellent negative predictive value of 0.97 aligning the LTA in abnormal collagen response (Sun et al., 2019). On the other hand, Berrou et al. have also reported a male patient with a gain‐of‐function *FLNA* variant and normal platelet count, but significant upregulation of several platelet activation steps when stimulated with ADP, suggesting that FLNA mutation heterogeneity correlates with different platelet functional impacts and points to opposite regulatory roles of FLNA in spreading and flow adhesion under shear (Berrou et al., 2013; Berrou et al., 2017).

## 5 CONCLUSION

*S*equencing of the *FLNA* gene should be considered in patients with periventricular nodular heterotopia, as carriers of pathogenic *FLNA* variants may be at an increased risk for both aortic dilatation and pulmonary disease, compatible with abnormalities in extracellular matrix interactions. We also suggest that patients with PVNH and pathogenic FLNA variants should routinely undergo full diagnostic work‐up for interstitial pulmonary disease, as extensive emphysematous lesions may be present in patients with mild or atypical symptoms. In addition, we suggest screening for pathogenic FLNA variants for all patients diagnosed with panlobular emphysema without history of smoking or alpha‐1‐antitrypsin deficiency. Pathogenic *FLNA* variants may affect platelet count and function, which may go undetected with routine laboratory tests. Immunofluorescence analysis of peripheral blood smears seems to be a useful tool in demonstrating the absent filamin in the novel variants.

## CONFLICT OF INTEREST

None declared.

## AUTHOR CONTRIBUTIONS

LMT, EL, TH, RL, and MP were involved in study design. LMT was involved in collection of clinical data, drafting, and submission of manuscript. SK was involved in immunofluorescence analyses. TH was involved in platelet functional analyses. KV was involved in radiographic images and their interpretation. LMT, SK, EL, TH, KV, RL, and MP were involved in critical review and revision of manuscript.

## FUNDING INFORMATION

The authors have not declared a specific grant for this research from any funding agency in the public, commercial, or not‐for‐profit sectors.

## PATIENT CONSENT FOR PUBLICATION

Obtained.

## Data Availability

The data that support the findings of this study are available from the corresponding author upon reasonable request.
